# Nurses’ Knowledge and Attitudes towards Biosimilar Medicines as Part of Evidence-Based Nursing Practice—International Pilot Study within the Project Biosimilars Nurses Guide Version 2.0

**DOI:** 10.3390/ijerph191610311

**Published:** 2022-08-19

**Authors:** Adriano Friganović, Wioletta Mędrzycka-Dąbrowska, Sabina Krupa, Ber Oomen, Nico Decock, Alessandro Stievano

**Affiliations:** 1 Department of Nursing, University of Applied Health Sciences, Mlinarska Cesta 38, 10000 Zagreb, Croatia; 2 Department of Anaesthesiology and Intensive Medicine, University Hospital Centre Zagreb, 10000 Zagreb, Croatia; 3 Department of Anaesthesiology Nursing & Intensive Care, Faculty of Health Sciences, Medical University of Gdansk, 80-211 Gdańsk, Poland; 4 Institute of Health Sciences, College of Medical Sciences, University of Rzeszow, 35-310 Rzeszow, Poland; 5 European Specialist Nurses Organization, 6821 HR Arnhem, The Netherlands; 6 Nurse Anaesthesia School, University Hospital of Lille, 111 Rue Charles Debierre, 59000 Lille, France; 7 Centre of Excellence for Nursing Scholarship OPI, Tor Vergata University of Rome, Via Cracovia 50, 00133 Rome, Italy

**Keywords:** biosimilars, pharmacists, knowledge, interchangeable products, health care, EBCNP

## Abstract

Introduction: The increasing availability of biosimilars can increase patient access to these drugs and reduce the economic burden. Nurses play a key role in the education, administration, pharmacovigilance and management of the side effects of biosimilars. The aim of this study was to assess the knowledge and attitudes of nurses towards biosimilar drugs in different countries. Methods: An international cross-sectional study was conducted from November 2021 to February 2022. The survey was carried out using Computer-Assisted Web Interview (CAWI), sent by the CAWI panel via the website. Results: The results showed that nurses with a greater level of education felt most knowledgeable about biosimilars (χ^2^ = 105.813, df = 2, *p* < 0.001). One-third of nurses with a doctorate and a second degree said biosimilars are used in their workplace (χ^2^ = 48.169, df = 4, *p* < 0.001); most nurses with a second degree said that they had never heard of biosimilars (41%). Doctorate-level nurses thought knowledge is the key factor to increasing biosimilar uptake (97%). Conclusions: Nurses are not knowledgeable about biosimilars. Most would like to participate in training on biosimilars. This is a very important topic, because biosimilars are constantly evolving in medicine.

## 1. Introduction

According to the definition by the European Medicines Agency (EMA), a biosimilar is a medicine with a very similar quality, safety and immunogenicity profile to already approved original biological medicines [1,2,3].

Biopharmaceuticals or biological products are medicinal products manufactured using living systems, i.e., genetically modified cells, bacteria, yeast or mammalian cells [1]. The most important distinguishing feature of biological drugs is the way they are produced; they are produced by living cells and therefore it is not possible to describe a detailed protocol for their production, which significantly distinguishes them from synthetic drugs. The vast majority of biological drugs have a complex structure and a very large molecule [4]. Such therapies play a key role in treating life-threatening and chronic conditions including cancer, diabetes, rheumatoid arthritis, and enteritis. [3]. Small-molecule drugs, on the other hand, are chemically synthesized, and different manufacturers can produce identical drugs, known as generic drugs [5].

A biosimilar medicine (BSM) is produced as a very similar copy of a previously registered innovative biological medicine [1]. A biosimilar drug is not a generic drug in pharmacological terms [5]. Approval of a biosimilar drug for use requires research to determine its structural similarity, research with the use of biological tests, pharmacokinetic studies, and clinical trials assessing the effectiveness and safety of the therapy. A biosimilar medicine must be bioequivalent, and its production, like the production of the original medicine, is strictly supervised by the regulatory agency [4,6].

It is important to also mention drugs that occupy an intermediate place between synthetic and biological, which are called “semi-synthetic drugs”. These are substances whose starting substrate has been isolated from biological material, but the correct molecule of the active substance has been created by in vitro chemical modifications of the original substrate. Good examples of this group of drugs are semi-synthetic penicillins or chemically modified preparations of hyaluronic acid or heparin [4].

The increasing availability of biosimilars can increase patient access to these drugs and reduce the economic burden [3]. Lack of knowledge of biosimilars can prevent their use, limiting their health benefits [1]. The reasons for the low use of biosimilars are multifaceted [2]. The main common features are the limited understanding of health care providers and patients about the development of biosimilar medicines, which may make it difficult to use them [6]. The introduction of biosimilar drugs is a process that is a natural market phenomenon. It brings many benefits for treatment, especially in connection with the possibility of lowering the prices of expensive biological drugs. In the future, advances in biotechnology will make it possible to simplify and reduce the cost of producing biological drugs, which will contribute to an even greater reduction in the cost of therapy [4].

Although biosimilars undergo rigorous assessment and direct comparison of the biosimilar with the original biologic shows that efficacy and side effects are similar, they are by no way a silver bullet of modern pharmacotherapy. There are challenges regarding development of a reproducible manufacturing process or challenges in demonstrating equivalence, safety and efficacy [7].

Nurses play an integral role in the education, administration, pharmacovigilance, and management of the side effects of biologics, and the use of biosimilars will bring new challenges for nurses in terms of education, treatment and patient monitoring [6]. Training nurses in new products is often ad hoc and incomplete; as a result, nurses may be unaware of the complexities and consequences of using biologics. Knowledge gaps result in inaccurate patient information, suboptimal application, and adverse events that can delay access and reduce the therapeutic benefit to the patient. Additional research on biosimilar medicines is required to identify current gaps in the knowledge and educational needs of nurses across Europe. Education about biosimilar medicines is critical to their successful incorporation into practice [8].

The aim of this study was to assess the knowledge and attitudes of nurses towards biosimilar drugs in different countries. Thanks to this study, the first in Europe, we may learn if biosimilar drugs have positive or negative reception among nurses, and whether there is knowledge deficit on this subject. This could in turn lead to a change in pre- and postgraduate education in nursing.

## 2. Materials and Methods

### 2.1. Study Design

An international cross-sectional study was conducted from November 2021 to February 2022.The survey was carried out using Computer-Assisted Web Interview (CAWI), sent by the CAWI panel via the website https://fonse.eu/blog/project-biosimilars-nurses-guide-version-2-0/ (accessed on 25 November 2021). A 21-question English-language survey was made available on nursing website. The authors followed the guidelines of the Helsinki Declaration (World Medical Association, 2013) and STROBE (Strengthening the Reporting of Observational Studies in Epidemiology) [9].

### 2.2. A Research Tool

A 21-item questionnaire was developed and adapted to the current use and regulations of biosimilars. The questionnaire consisted of three parts: (1) demographic and general information of the respondents; (2) the respondents’ knowledge of biosimilar medicines; (3) the respondents’ attitude to issues related to biosimilar medicines (Supplementary Materials).

### 2.3. Participants

The questionnaire was posted on the website of the European Specialist Nurses Organization (ESNO). A total of 866 nurses from 11 countries participated in this study. All the respondents worked in medical facilities.

### 2.4. Bioethical Commission

This study was conducted in accordance with the Declaration of Helsinki, suggested by the Scientific Committee and approved by the European Specialist Nurses Organization Board of Directors and General Assembly at its meeting on 24 November 2021. The research team respected and integrated the ethical standards during the investigation processes, particularly during the collection and analysis of the qualitative and quantitative data. Information strictly required for the purpose of this study was collected in the form of anonymized data.

### 2.5. Statistics

The statistical analysis was carried out using SPSS software v25.0.0 (IBM Corp. Released 2017. IBM SPSS Statistics for Windows, Version 25.0. Armonk, NY, USA: IBM Corp.). Qualitative variables were presented by means of counts and percentages, and the quantitative variable was characterized by means of an arithmetic mean and standard deviation. The significance of differences between more than two groups was checked by the Kruskal–Wallis test (in the case of obtaining statistically significant results, Bonferroni post hoc tests were used) and between the two groups by the Mann–Whitney U test and Student’s *t* test. In order to find the relationship of strength and direction between the variables, a correlation analysis was used to calculate Spearman’s correlation coefficients. Chi square tests were used for qualitative variables. A *p*-value below 0.05 was considered to be of statistical significance.

## 3. Results

### 3.1. Characteristics of the Study Group

A total of 866 nurses participated in this research. The majority of this sample were female nurses (578–66.7%). The average age was 41–50 years. The gender, age, country of employment, work experience, specialization and field of expertise of the participants are shown in detail in Table 1.

### 3.2. Respondents’ Knowledge of Biosimilars

Knowledge about biosimilars was assessed by 66.3% of respondents at the beginner level, 12.1% at the competent level, and 21.6% at the advanced beginners. Doctorate-level and Bachelor’s degree-level nurses felt most knowledgeable about biosimilars (χ^2^ = 105.813, df = 2, *p* < 0.001).

A total of 40.8% of the respondents learned about biosimilar medicines from the internet—mainly Anesthesia and Intensive Care nurses and Infection Control nurses. A total of 31.2% of respondents have never heard of biosimilar drugs. Only 3.9% of the respondents participated in training on biosimilar medicines. A total of 68.4% of the respondents did not know the benefits of using biosimilars in their field and 36.8% did not know whether biosimilars are used in their workplace. One-third of doctorate and Master’s degree nurses said biosimilars are used in their workplace (χ^2^ = 48.169, df = 4, *p* < 0.001); most Master’s degree nurses said that they did not know (41%).

The analysis showed that men rated their knowledge of biosimilars significantly higher than women (Z = 7.98; *p* < 0.001). In the age group > 60, knowledge was rated the highest than in the other age groups, while respondents aged 51–60 assessed their knowledge significantly lower than respondents aged 20–30, 31–40, 41–50 and >60 years. There were no statistically significant differences between the groups of 20–30 and 41–50 years (H (4) = 57.19; *p* < 0.001). The amount of their knowledge was best rated by respondents from the Netherlands, North Macedonia, Pakistan and Bosnia and Herzegovina, significantly higher than respondents from other countries. The subjective assessment of knowledge among respondents from Belgium, Croatia, the United Kingdom, Finland, Italy, Malta and Poland was assessed at a comparable level (H (10) = 525.94; *p* < 0.001). Respondents who completed Master’s degree studies rated their knowledge higher than the respondents, with doctoral education and after Bachelor’s degree studies (H (2) = 274.69; *p* < 0.001). A positive, statistically significant correlation was obtained between the subjective assessment of knowledge and work experience, which in the interpretation means that with the increase in work experience, the assessment of knowledge about biosimilars increases (rHO = 0.18; *p* < 0.001). Respondents with specializations in Anesthesiology and Intensive Care, Infection Control, Clinical Nutrition, Wound Healing and Neurology rated their knowledge of biosimilars the lowest (no differences between these groups). Knowledge was rated the highest among respondents with a specialization in Psychiatry—significantly higher than in the other groups. Subjects with specializations in Oncology and Surgical Nursing and people without a specialization assessed their knowledge to a comparable degree, significantly higher than in the other groups—apart from Oncology (H (8) = 338.06; *p* < 0.001). Among the respondents working in the Emergency Department, Infection Control, Clinical Nutrition, Dermatology, Neurology, Palliative Care, and the Trauma Center and people without a job rated their knowledge of biosimilars lowest. The level of knowledge at a highest level was significantly higher among the respondents who worked in the Psychiatric and Neonatology wards than in the other groups. Respondents working in Anesthesiology and Intensive Care, Surgical Nursing, Oncology, as Charge Nurses and in eHospitals assessed their knowledge of biosimilar medicines higher than in the other groups—except for those in Neonatology and Psychiatric (H (14) = 474.77; *p* < 0.001). A detailed summary of the results is presented in Table 2.

### 3.3. Respondents’ Attitude to Biosimilars

Most nurses (64%) did not talk to patients about biosimilars due to a lack of knowledge, and only 4.5% of respondents talked to patients about biosimilars. The majority of nurses (85.8%) believed that patients should be informed about the treatment options for biosimilars, while 61.9% of respondents believed that nurses should inform patients about biosimilars. Characteristics of the study group: 87.2% of the respondents would be willing to take part in training about biosimilars; 85.8% of nurses believed that patients should be informed about the possibilities of treatment with biosimilar drugs, but the surveyed nurses avoided talking to patients about biosimilar drugs due to a lack of knowledge; nurses working in perioperative care believed that doctors were best prepared to provide information about biosimilar medicines; wound care nurses believed other health care professionals should so inform patients, but subjects in other specializations felt nurses were best suited to do so.

The analysis showed that men more often than women believe that the use of biosimilars is the future, while women more often do not have an opinion on this subject (χ^2^ (1) = 6.63; *p* < 0.05). The respondents aged 20–30 and 41–50 most often believed that the use of biosimilar drugs is the future, while respondents aged > 60 years most often had no opinion on this subject (χ^2^ (4) = 114.70; *p* < 0.001). The respondents from Bosnia, Finland, Italy, North Macedonia and Pakistan most often believed that the use of biosimilar drugs is the future, and respondents from Belgium, England, Malta, the Netherlands and Poland most often had no opinion on this subject (actual value > expected value) χ^2^ (10) = 367.25; *p* < 0.001. In the group of respondents with doctoral education or a Bachelor’s degree, the use of biosimilars was most often considered to be the future (actual value > expected value), while respondents who completed a Master’s degree most often did not have an opinion on this subject (χ^2^ (2) = 164.74; *p* < 0.001). Statistically significantly more often respondents whose work experience was up to 17 years believed that the use of biosimilar drugs was the future, while respondents with more than 20 years of work experience more often did not have an opinion on this subject (t (863) = 4.23; *p* < 0.001). In fact, most frequently respondents with specializations in Wound Healing, Neurology, Oncology and those who had no specialization believed that the use of biosimilars was the future (actual value > expected value). On the other hand, respondents with Infection Control, Clinical Nutrition and Psychiatry specializations most often did not have an opinion on this subject (χ^2^ (8) = 176.53; *p* < 0.001). Most often respondents who worked in Emergency, Charge Nurse, Dermatology, eHospital, Neonatology, Neurology and the Trauma Center, or those who were unemployed, believed that the use of biosimilar medicines was the future (actual value > expected value). In contrast, respondents working in Surgical Nursing, Oncology, Infection Control, Psychiatric Ward, Clinical Nutrition and Palliative Care most often did not have an opinion on this subject (χ^2^ (14) = 335; *p* < 0.001). A detailed summary of the results is presented in Table 3.

## 4. Discussion

This study provided an overview of the knowledge and attitudes of nurses towards biosimilar medicines in different European countries in 2022. Thanks to this study, the first in Europe, we learned that despite the positive reception of biosimilar drugs among nurses, another professional group has an evident knowledge deficit on this subject. This could lead to a change in pre- and postgraduate education in nursing, especially since in most European countries, nurses—to a greater or lesser extent—can prescribe prescription drugs. Nurses, as among the leading groups of health care workers, face the challenge of introducing biosimilar drugs into clinical practice. This research shows that nurses with a Bachelor’s degree have the greatest knowledge about the advantages of biosimilar drugs, while the general knowledge is low. A study by Peipert et al. in the United States examined the knowledge of 269 oncologists. Despite the high prevalence of biosimilars, knowledge of the basic features of biosimilars was low, and the oncologists in environmental and private practice were more often concerned about their safety and efficacy [10]. In the study by Guliani et al. carried out in 2017 through the European Society for Medical Oncology (ESMO) in Spain, 393 oncologists were examined, and their general knowledge about biosimilars was assessed as medium or very high. There were some differences in responses between prescribers in Europe and the Asia-Pacific region with regard to the knowledge and convenience of use of biosimilar medicines; a higher rate of biosimilar drug use in routine clinical practice was found in the Asia-Pacific region than in Europe [11]. In a study by Poon et al., it was shown that the current level of medical knowledge of biosimilar medicines in Taiwan is low. The higher the knowledge score, the greater the confidence in biosimilar medicines, and the greater the knowledge of the relevant regulations.

In a review by Leonard et al., US and European health care providers were shown to continue to be cautious about biosimilar medicines, citing limited knowledge of biosimilars, low comfort on the part of doctors in prescribing them, and concerns about safety and efficacy [12]. In order to achieve the cost savings potential of biosimilars, education of clinicians will be necessary to fill the gaps in their knowledge [13]. Gökdemir et al. showed that the general knowledge of doctors about biosimilar medicines was low, and that they had doubts about the effectiveness and safety of biosimilar medicines, which may be related to the lack of knowledge. Organized training programs in this area may raise the level of knowledge and positively influence the opinions and attitudes of doctors towards biosimilar medicines [14]. A cross-sectional study by Demir-Dora et al. of 228 medical students showed that the medical students had insufficient knowledge about biosimilar medicines. The researchers suggested that in order to develop a positive attitude towards prescribing biosimilar medicines, knowledge of biological and biosimilar medicines should be given to doctors early in their medical studies by including this topic in the medical school curriculum [15].

One-third of nurses in doctoral studies and graduate studies said that biosimilars were used in their workplace, while most nurses in graduate studies say they had no knowledge of them being used. In comparative studies on the use of biosimilars conducted by Barszczewska et al. it was shown that 54 biosimilar drugs are registered in Poland and France, 50–60% of which are reimbursed by insurance to the patients [16]. In a study by Dean et al., practice setting and hospital ownership status were shown to have the greatest association with biosimilar drug use, suggesting that practices play a role in referring physicians to certain drugs. The authors also emphasized that biosimilars can be successfully used in the conditions of a hospital outpatient clinic or doctor’s surgery [17].

Our research showed that nurses with a Bachelor’s degree and nurses with a Ph.D are convinced that biosimilar medicines are the future, while no one opposed their usefulness. A study by Kim et al. showed that physicians’ full understanding of biosimilars and carefully thought-out communication strategies can help support patients starting or switching to biosimilars [18].

The largest number of surveyed nurses at all levels of education avoided talking to patients about biosimilars because they knew nothing about them. Nurses at all levels of education agreed that patients should be informed about treatment options with biosimilar medicines. In a review by Vandenplas et al., several large questionnaire studies showed a lack of knowledge and confidence in biosimilar medicines in European patients in recent years. It is therefore most important to establish close cooperation between all interested parties to communicate, develop and disseminate factual information on biosimilar medicines to patients [19].

Nurses with a lower level of education would be more likely to attend training courses on biosimilar drugs. While an improvement in knowledge has been observed over time, the literature reports generally low to moderate levels of awareness, knowledge and confidence in biosimilars in health care professionals and patients. A study by Barbier et al. advocated a structured, multistakeholder framework at both European and national level to improve understanding and acceptance of biosimilar preparations. It also proposed a series of practical recommendations that could help shape policy and guide stakeholders, which could contribute to the health system benefits of biosimilar competition [20].

The internet was the main source of information, education and training on biosimilars for nurses. In a study by Frantzen et al. only 43% out of 629 respondents knew what biosimilars were. In that study, the main sources of information were rheumatologists and patient associations [21].

In the future, nurses will have the opportunity to play a key role in the new era of drugs in the area of administration and education. Due to the complexity of biosimilars, education of nurses with regard to these products should be provided for effective pharmaceutical management in hospital settings. In addition, it should be emphasized that well-educated nurses can be a very significant link in the interdisciplinary team, because they are usually the first to notice possible adverse effects or improvements in the patient’s condition following the use of biosimilars [22].

Biosimilars are not used in most medical centers. However, if they are used, nurses are not informed about it (according to the results of the study), which might expose patients to some risks, immunogenicity being among the most significant [23]. In addition, large percentage of nurses do not have any training or knowledge about biosimilar medicines. Despite the great interest and willingness to participate in trainings, decision makers do not organize them on this topic.

Our research shows that the type of specialization and the department in which the respondents work most correlate with the knowledge about biosimilars or the belief in their importance in the future. Most biosimilar preparations are registered in such areas as oncology or dermatology and nurses working in those fields turned out to be most knowledgeable about these drugs. Biosimilars have been on the market for approximately 1.5 decades [23,24]. An astonishing fact is that in our study, older nurses declared greater knowledge about these drugs, even though they certainly did not get this knowledge from their studies’ curriculum. It might simply be because of the clinical experience of these individuals.

## 5. Study Limitations

Very little research has been performed on biosimilar medicines, and ours is the first survey of nurses of various specialties in different countries on that subject. Consequently, there were no standardized tools to be used and because of rather narrow scope of some questions, our study may have a limited value. As it was an online survey, this led to significant differences in the number of responders from individual countries so the research may not be fully representative.

## 6. Conclusions

Biosimilars are a new topic in the literature, but a very important one. Nurses evaluate their knowledge of biosimilars as quite low and do not know their actions. Nurses learn about biosimilars mainly from the internet. In conclusion, biosimilars are not a popular topic among nurses. It is necessary for them to be educated on this subject, because this lack of knowledge could have serious consequences if biosimilars were introduced to a ward where the nurses do not understand them.

## 7. Implication for Practices

Globally, efforts need to be made to align the terms, definitions and guidelines for the development and approval of biosimilar medicines. Biosimilar drugs should be of interest to future educational initiatives. There is a high demand for further educational activities, with equal preferences for online and face-to-face initiatives. Nurses should take the time to learn more about biosimilar medicines to educate patients and work with other health care professionals to make these medicines more readily available to those who may benefit from them.

## Figures and Tables

**Table 1:** Sociodemographic factors (N = 866).

		N	%
**Gender**	Male	288	(33.3%)
	Female	578	(66.7%)
**Age**	20–30 years old	206	(23.8%)
	31–40 years old	201	(23.2%)
	41–50 years old	307	(35.5%)
	51–60 years old	80	(9.2%)
	Over 60 years old	72	(8.3%)
**Country of work**	Croatia	205	(23.7%)
	Malta	108	(12.5%)
	Italy	89	(10.3%)
	United Kingdom	88	(10.2%)
	Bosnia and Herzegovina	73	(8.4%)
	Poland	70	(8.1%)
	Finland	42	(4.8%)
	Pakistan	39	(4.5%)
	North Macedonia	73	(8.4%)
	Belgium	38	(4.4%)
	Netherlands	41	(4.7%)
**Experience in profession**	≤5 yrs	150	(17.3%)
	6–10 yrs	100	(11.5%)
	11–15 yrs	195	(22.5%)
	16–20 yrs	61	(7.0%)
	21–25 yrs	141	(16.3%)
	26–30 yrs	73	(8.4%)
	31+ yrs	146	(16.9%)
**Education**	Master’s degree studies	573	(66.2%)
	Bachelor’s degree studies	179	(20.7%)
	Doctorate	114	(13.2%)
**Field of expertise**	No Specialization	299	(34.5%)
	Surgical Nursing	217	(25.1)
	Infection Control	114	(13.2%)
	Anaesthesiology and Intensive Care	73	(8.4%)
	Neurology	39	(4.5%)
	Wound Healing	38	(4.4%)
	Psychiatry	37	(4.3%)
	Clinical Nutrition	28	(3.2%)
	Oncology	21	(2.4%)
**Department**	Anaesthesiology and Intensive Care	224	(27%)
	Oncology	107	(12.9%)
	No Job	70	(8.5%)
	Emergency	60	(7.2%)
	Surgical Nursing	56	(6.8%)
	Infection Control	39	(4.5%)
	Neurology	39	(4.7%)
	Trauma Center	39	(4.7%)
	eHospital	39	(4.7%)
	Dermatology	38	(4.6%)
	Psychiatry	37	(4.5%)
	Neonatology	34	(4.1%)
	Palliative Care	34	(4.1%)
	Clinical Nutrition	28	(3.4%)
	Charge Nurse	22	(2.7%)
**Years of work in the profession** (*Min* = 1–*Max* = 43)		M * = 18.2	SD ** = 11.01

* M = mean; ** SD = standard deviation.

**Table 2:** The level of knowledge about biosimilar drugs vs. sociodemographic data.

*Variables*		Level on Biosimilar Knowledge (* 1 = Novice–3 Competent)			Statistic	*p* Value
		N	Mean	SD		
**Gender**	Female	578	1.31	0.57	7.98	0.000 ^a^
	Male	288	1.74	0.82		
**Age**	20–30	206	1.35	0.5	57.19	0.000 ^b^
	31–40	201	1.46	0.51		
	41–50	307	1.51	0.82		
	51–60	80	1.03	0.24		
	>60	72	1.95	0.99		
**Country**	Belgium	38	1.02	0.16	525.94	0.000 ^b^
	Bosnia and Herzegovina	73	1.99	1		
	Croatia	205	1.45	0.49		
	United Kingdom	88	1.01	0.1		
	Finland	42	1	0		
	Italy	89	1.03	0.18		
	Malta	108	1	0		
	Netherlands	40	2.89	0		
	North Macedonia	73	2.46	0.5		
	Pakistan	39	2	0		
	Poland	70	1.12	0.33		
**Education**	Master’s degree studies	179	2.19	0.72	274.69	0.000 ^b^
	Bachelor’s degree studies	573	1.31	0.58		
	Doctorate	114	1	0		
**Field of expertise**	No specialization	299	1.66	0.67	338.06	0.000 ^b^
	Anesthesiology and intensive care	73	1	0		
	Surgical nursing	217	1.57	0.75		
	Infection control	114	1	0		
	Clinical nutrition	28	1	0		
	Psychiatry	37	3	0		
	Wound healing	38	1	0		
	Neurology	39	1	0		
	Oncology	21	2	0		
**Department**	No job	33	1	0	474.77	0.000 ^b^
	Anesthesiology and intensive care	224	1.62	0.73		
	Surgical nursing	56	1.57	0.68		
	Emergency	60	1	0		
	Oncology	107	1.69	0.86		
	Infection control	39	1	0		
	Psychiatric ward	37	3	0		
	Charge nurse	22	2	0		
	Clinical nutrition	28	1	0		
	Dermatology	38	1	0		
	eHospital	39	2	0		
	Neonatology	34	3	0		
	Neurology	39	1	0		
	Palliative care	34	1	0		
	Trauma center	39	1	0		
Years of work in the profession					rHO	*p* value
					0.18	0.000 ^c^

SD = standard deviation; * ^a^ = Mann–Whitney’s test; ^b^ = Kruskal–Wallis test; ^c^ = Spearman’s correlation test; *p*—level of significance; rHO—Spearman’s rank correlation.

**Table 3:** Attitude to biosimilars vs. sociodemographic data.

Variables		Attitude to Biosimilars			Statistic	*p* Value
		I Think They Are the Future		I Have No Opinion about It		
**Gender**	Female	308		270	6.63	0.010 ^a^
	Male	180		108		
**Age**	20–30	144		62	114.7	0.000 ^a^
	31–40	96		105		
	41–50	204		103		
	51–60	41		39		
	>60	3		69		
**Country**	Belgium	4		34	367.25	0.000 ^a^
	Bosnia and Herzegovina	70		3		
	Croatia	125		80		
	United Kingdom	9		79		
	Finland	38		4		
	Italy	76		13		
	Malta	40		68		
	Netherlands	2		35		
	North Macedonia	70		3		
	Pakistan	37		2		
	Poland	15		55		
**Education**	Master’s degree studies	172		7	164.74	0.000 ^a^
	Bachelor’s degree studies	242		331		
	Doctorate	74		40		
**Field of expertise**	No specialization	204		95	176.53	0.000 ^a^
	Anesthesiology and intensive care	37		36		
	Surgical nursing	107		110		
	Infection control	42		72		
	Clinical nutrition	1		27		
	Psychiatry	2		35		
	Wound healing	37		1		
	Neurology	38		1		
	Oncology	20		1		
**Department**	No job	32		1	335.63	0.000 ^a^
	Anesthesiology and IC	112		112		
	Surgical nursing	22		34		
	Emergency	37		23		
	Oncology	41		66		
	Infection control	2		37		
	Psychiatric ward	2		35		
	Charge nurse	21		1		
	Clinical nutrition	1		27		
	Dermatology	37		1		
	eHospital	37		2		
	Neonatology	34		0		
	Neurology	38		1		
	Palliative care	1		33		
	Trauma center	37		2		
Years of work in the profession		N	* M	** SD	Statistic	*p* value
		488	16.71	11.73	4.23 ^a^	0.000 ^b^
		377	20.14	11.89		

* M = mean; ** SD= standard deviation; ^a^ = chi square test; ^b^ = Student’s *t* test; *p*—level of significance.

## Data Availability

The authors declare that the data from this study are available from the author S.K. upon request.

## Supplementary Materials

The following supporting information can be downloaded at: https://www.mdpi.com/article/10.3390/ijerph191610311/s1.
