# Supplementary material for: Nurses’ Knowledge and Attitudes towards Biosimilar Medicines as Part of Evidence-Based Nursing Practice—International Pilot Study within the Project Biosimilars Nurses Guide Version 2.0
Source: Int J Environ Res Public Health. 2022 Aug 19;19(16):10311. doi: 10.3390/ijerph191610311 (PMC9408045; doi:10.3390/ijerph191610311)
Supplement: Supplementary file 1 [file ijerph-19-10311-s001.zip › ijerph-1815303-supplementary.pdf]

Nurses' knowledge about biosimilars

Q1. Age………

Q2. Gender…….

Q3. Years of work in the profession…..

Q4. The country I currently work…..

Q5 Education…….

Q6.My field of expertise……

Q7. Where did you first hear and learn about biosimilars?

- Internet
- Friends
- Journals
- Social Media
- Other……..
- I've never heard about biosimilars

Q8. My level on Biosimilar knowledge is

- Novice Advanced Beginner Competent

Q9. What were the main sources of information, education or training you encountered?

- In my place of work
- Conferences
- Courses
- Online lecture
- I did not participate

Q10. Do you know what are the advantages of using biosimilars in your field

- Yes No

Q11. Are biosimilars used in your workplace?

- Yes No I don't know

Q12. What factors could increase biosimilar uptake ? (In your opinion)

- Knowledge
- Access to biosimilars
- Others, name which …………………………………… ..

Q13 What is your attitude towards biosimilar medicines

- I think they are redundant
- I think they are the future
- I have no opinion about it

Q14. How do you communicate with your patients about biosimilars? You can choose more than one answer

- I explain how they work
- I discuss the side effects of biosimilars with patient
- I describe examples of use in a given field of medicine
- I don't talk with the patient about biosimilars because I don't know anything about it

Q15. Do you know the names of biosimilars? If YES, please name at least three

- Yes, for example………………………………………………
- No, I don’t know

Q16. Do you have any courses about biosimilars in your workplace?

- Yes No

Q17. Have you come across the topic of biosimilars in the literature?

- Yes No

Q18. Do you think that patients should be informed about the treatment options with biosimilars?

- Yes No I don’t have an opinion about it

Q19. Who do you think is best placed to provide the education about biosimilars?

- Doctors Nurses Other heathcare workers

Q20. Would you like to attend training about biosimilars?

- Yes No

Q21.If you have any suggestions for biosimilars and education, feel free to post here
